# Mapping the scientific landscape of robotic hernia repair: a bibliometric and topic modeling analysis of thematic transitions

**DOI:** 10.1007/s11701-026-03534-y

**Published:** 2026-06-19

**Authors:** Yongxuan Yuan, Liqun Wang, Lierui Chen, Qinpei Ke, Kangni Chen, Zhiyang Li, Jiehua Zheng

**Affiliations:** Department of Thyroid, Breast and Hernia, The Second Affiliated Hospital, Shantou University Medical College, No.69 North Dongxia Road, Shantou, 515041 Guangdong P.R. China

**Keywords:** Robotic surgery, Hernia repair, Bibliometric analysis, LDA model

## Abstract

Robotic hernia repair (RHR) has profoundly transformed modern surgery, yet it remains intensely debated. To understand this dynamic field, bibliometric analysis offers a powerful tool to evaluate its evolving research trends. Our study presents a comprehensive bibliometric and machine learning analysis of 1,503 publications from 2003 to 2025. The literature demonstrates a growth trend (R^2^ = 0.96), characterized by initial latency (2003–2015), steady adoption (2016–2020) and a recent exponential explosion (2020–2025) peaking at 262 publications in 2025. The United States is the leading contributing nation (*n* = 892), followed by Italy (*n* = 98), Germany (*n* = 71), and China (*n* = 68). Key contributors include Kudsi OY, Malcher F, and Muysoms F, with Surgical Endoscopy serving as the predominant publication venue (*n* = 203). Spatial and relational networks were mapped using VOSviewer and CiteSpace. Critically, Latent Dirichlet Allocation (LDA) topic modeling combined with principal component analysis (PCA) stratified the corpus into four major research domains comprising the anatomical spectrum, the balance between resource and risk management, hardware evolution and surgical education, and technical complexity coupled with high-level evidence. Longitudinal regression of thematic probabilities revealed a significant thematic transition in research emphasis from early hardware feasibility and technical exploration toward value-based evaluation. Current research priorities are increasingly focused on rigorous cost-effectiveness evaluation in high-risk populations together with the generation of high-quality randomized evidence. In conclusion, this multidimensional analysis illustrates how this field is increasingly guided by the interconnected domains of Evidence, Education and Economics, which align with established frameworks for sustainable surgical progress.

**Supplementary Information:**

The online version contains supplementary material available at 10.1007/s11701-026-03534-y.

## Introduction

Repair of abdominal wall and inguinal hernias is among the most frequently performed surgical interventions worldwide. According to global estimates, the annual global volume of hernia repair procedures has exceeded 20 million cases [1–3]. Hernia surgery has undergone several major technological iterations and paradigm shifts over the past 2 decades, evolving from traditional tissue-based suture repair to the tension-free placement of synthetic mesh represented by the Lichtenstein repair and subsequently to minimally invasive revolution, exemplified by intraperitoneal onlay mesh repair (IPOM), totally extraperitoneal repair (TEP) and transabdominal preperitoneal repair (TAPP). The widespread adoption of these minimally invasive modalities has significantly reduced surgical site infection rates, mitigated postoperative pain and accelerated patient recovery [2, 4]. Nevertheless, with the growing demand to reconstruct extreme challenging presentations including complex abdominal wall defects, giant incisional hernias and loss of domain, the limitations of conventional laparoscopy have become progressively more apparent, especially in suboptimal ergonomics, restricted two-dimensional visualization and constrained degrees of freedom imposed by rigid instrumentation [5].

In this context, robotic surgery has emerged as a disruptive innovation in general surgery, triggering a new revolution in abdominal wall reconstruction. Empowered by high-definition of three-dimensional stereoscopic visualization, physiological tremor filtration and wristed instrumentation with seven degrees of freedom, the robotic platform confers substantial theoretical advantages [6, 7]. These advantages are particularly pronounced in executing meticulous dissection, complex suturing and the fixation of large prostheses within confined anatomical space, such as the retrorectus and preperitoneal planes [2].

Macroscopic clinical data underscored that the proportion of robotic hernia repair (RHR) among all hernia procedures in the United States surged significantly from 2.1% to 21.9%. This rapid proliferation is aggressively replacing the conventional paradigms of open and laparoscopic approaches [8]. Concurrent with the widespread adoption of this technology, advanced abdominal wall reconstruction techniques, represented by robotic enhanced-view totally extraperitoneal (r-eTEP) repair and robotic transversus abdominis release (rTAR) have been rapidly developed [9, 10].

However, the rapid expansion of robotic surgery has been accompanied by intense academic debate within the global surgical community. Supporters argue that robotic surgery enables the conversion of complex abdominal wall reconstructions which required open surgery into minimally invasive surgery, thereby substantially enhancing the postoperative quality of life [11, 12]. Conversely, objectors point out that robotic platforms impose massive capital cost, heavy expenditure on instruments and prolonged operation time. In addition, without robust evidence from large randomized controlled trials, the application of robotic platform for anatomically simple inguinal hernia repair fails to provide additional clinical benefits. Instead, it exacerbates the inefficient utilization of medical resources [13–15]. Furthermore, the steep learning curve of RS exposes surgeons in the early stage of training to increased intraoperative burden and may potentially contribute to a higher long term risk of recurrence [16].

Under this highly fragmented academic background, the global literature on RHR has grown explosively over the past decade. This expansion encompasses dimensions ranging from reports of single center and data mining of national registries to health economic evaluations of cost-effectiveness. However, massive and heterogeneous literature has substantially obscured the underlying knowledge architecture, collaborative network and latent trajectory of thematic evolution in this field. Traditional bibliometric analysis frequently remain limited to superficial level without penetrating the textual surface to capture the deep paradigm shifts that drive the evolution of this field. To bridge this gap, our study aims to conduct a comprehensive assessment of the global literature on RHR. This is achieved by integrating multidimensional bibliometric network mapping with Latent Dirichlet Allocation (LDA), an advanced approach for topic modeling in natural language processing. In this study, we will not only systematically reveal the global research landscape of RHR, but will also focus on isolating the 4 major thematic camps dominating current clinical decisions making. Combined with longitudinal regression analysis, we identified the transition of RHR from a technology driven phase toward value based health care, evidenced based standardization and reformation of surgical training. The conclusions of this study are anticipated to serve as a valuable resource for researchers new to RHR and provide rigorous, actionable guidance for subsequent mechanistic research and clinical trial planning.

## Materials and methods

### Search strategy and data cleaning

We conducted a literature search on the Web of Science Core collection (WoSCC) and Scopus. Both databases are widely used for scientific literature retrieval. The literature search was conducted on February 24, 2026. The search query employed in WoSCC was as follows: TS= ((robot* OR “robot-assisted” OR “robotic-assisted” OR “da vinci”) AND ( (hernia* NEAR/3 repair*) OR hernioplast* OR herniorrhaph* OR (“mesh” NEAR/3 hernia*))). Similarly, the search query applied in Scopus was: TITLE-ABS-KEY ((robot* OR “robot-assisted” OR “robotic-assisted” OR “da vinci”) AND ((hernia* W/3 repair*) OR hernioplast*OR herniorrhaph* OR (mesh W/3 hernia*) OR (hernia* W/3 mesh))). The time span was restricted to publications between January 1, 2003 and December 31, 2025. Only articles and reviews published in English were retained. Other document types such as editorial materials, letters and meeting abstracts were excluded. The initial literature retrieval yielded a total of 1371 records from Scopus and 1028 records from WoSCC. 37 records from Scopus and 23 records from WoSCC were excluded due to the absence of a DOI.The remaining records from both databases were merged into a unified document. Subsequently, we performed a deduplication process by using an automated DOI-matching approach. This process removed 850 overlapping records, and a cohort of 1503 unique documents was retained for the final bibliometric and visual analysis ultimately.

### Bibliometric analysis

For the 1503 included documents, we conducted a systematic bibliometric assessment by utilizing the R programming language as the primary platform and supplemented by VOSviewer and CiteSpace for specialized network mapping.

The R software environment (version 4.3.1) and its specialized “bibliometrix” package were employed to execute the bibliometric analysis. This platform was used to extract productivity metrics, plot annual growth trajectories of publications and generate the visualizations of temporal trends and leading academic contributors [17].

VOSviewer (version 1.6.20) was utilized to map the spatial and relational knowledge structure. It was applied to perform bibliographic coupling analysis of literature and to visualize institutional collaboration networks. By leveraging its distance-based mapping algorithms, distinct thematic clusters and collaborative communities were identified [18, 19].

CiteSpace (version 6.4.R1) was used for bibliometric analysis and visualization. Specifically, the burst detection algorithm was applied to identify institutions which experienced a significant surge in publication activity. Additionally, it was also utilized to construct a dual-map overlay of journals. The map showed citing journals on the left as research frontiers and cited journals on the right as intellectual base, which helped us track how knowledge flows from one topic to another [20].

### Topic modeling

To uncover latent thematic structures within the literature, we applied LDA modeling [21]. Bibliographic data comprising titles, abstracts and author keywords were extracted and combined. To ensure high-quality text representation, documents were retained only when an abstract was available and at least 20 valid tokens remained after text preprocessing.

The LDA model was based on the canonical latent Dirichlet allocation framework and executed using Gibbs sampling via the “topicmodels” R package [22, 23]. Candidate models with k values of 6, 8, 10 and 12 were compared under different Dirichlet prior settings. Model selection integrated quantitative diagnostics, including perplexity, C_v coherence, UMass coherence, topic granularity and clinical interpretability (Supplementary Tables 1 and 2). This combined strategy was used because improvement in statistical fit does not always indicate a more interpretable topic structure. Hyperparameter and random-seed sensitivity analysis were further performed to assess model robustness (Supplementary Tables 3 and 4). The final configuration was chosen as k = 8, α = 0.10 and β = 0.10. The top 10 high-weight terms from each generated topic were manually reviewed to assign thematic labels. A linear regression model was performed with year as the independent variable and mean topic probability as the dependent variable. By using relative probability rather than absolute publication counts, this approach accounts for the exponential growth in total publication volume. The significance and directionality of the slope were interpreted to characterize the thematic growth trends. To explore multidimensional relationships linking documents and topics, principal component analysis (PCA) was conducted on the document-topic distribution matrix θ, followed by construction of a Husson-Jongmans biplot (HJ-Biplot). This visualization was used to display document scores and topic vectors in two-dimensional principal component plane, thereby illuminating inter-topic relationships and highlighting representative documents.

## Results

### Literature growth trend

**Fig. 1:**
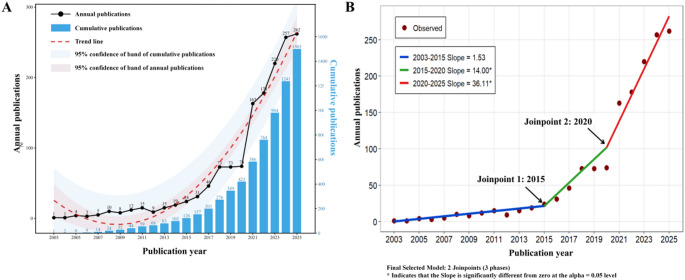
Distribution and Trends in Robotic Hernia Repair Research.** A** Publication output distribution and trends over time. The red dashed line represents the trend line (Trend line: y = 1.0335 × ^2^-11.8881x + 25.58). **B** Phases of publication output in robotic hernia repair. Asterisk indicates that the slope is significantly different from zero at the α = 0.05 level. Final selected model: 2 joinpoints

Conducting productivity analysis in a research field helps in understanding the dynamics and emerging trends within that field. The earliest related study in RHR was published in 2003, and after over 20 years of development, the cumulative output has reached 1503 publications. The annual output experienced gradual early accumulation and recently peaked at 262 publications in 2025, representing 17.43% of the total volume. The red dashed line in Fig. 1A represents the fitted trend line, showing an overall strong upward trajectory (R^2^ **=** 0.96). To determine the most appropriate growth model, we conducted a sensitivity analysis comparing joinpoint models with different numbers of turning points (Supplementary Table 6). The two-joinpoint model showed the lowest Bayesian Information Criterion (BIC = 192.57) and was therefore selected as the final model. This model estimated two breakpoints at 2014.76 (95% CI: 2012.76–2016.76) and 2019.52 (95% CI: 2018.17–2020.86). These continuous estimates were rounded to define the phase boundaries of 2015 and 2020 in Fig. 1B. The estimated slopes increased from 1.53 during 2003 to 2015 to 14.00 during 2016 to 2020 and 36.11 during 2021 to 2025. The latter two slopes were significantly different from zero at α = 0.05, indicating marked acceleration in publication growth after 2015 and a further increase after 2020.

Based on publication volume and slope changes, the research output in this field can be divided into three distinct phases. The first stage (2003 to 2015) had a total of 126 publications (8.38% of the total), which represents an initial nascent phase with steady output. The second stage (2016 to 2020) saw 297 publications (19.76% of the total). In the third stage (2020 to 2025), the field entered a period of exponential explosion (71.86% of the total). The trend line presented in Fig. 1A predicts continued and robust growth in scientific output for the foreseeable future.

### Country analysis

Analyzing national output and collaboration patterns provides a macroscopic understanding of global progress and emerging cooperative trends in RHR. The top ten most productive countries collectively contributed 1427 studies (94.94% of the total), accounting for nearly all global output. As detailed in Table 1, among these elite countries, five are located in Europe, three in Asia, and two in the Americas. The United States ranked first in publication output with 892 articles (59.35%), citation count with 12,914 citations (67.06%) and number of international partnerships with 60 partner countries (83.33%). Notably, almost all of these high-output countries are ranked among the top 25 globally by their projected 2025 gross domestic product.

**Fig. 2:**
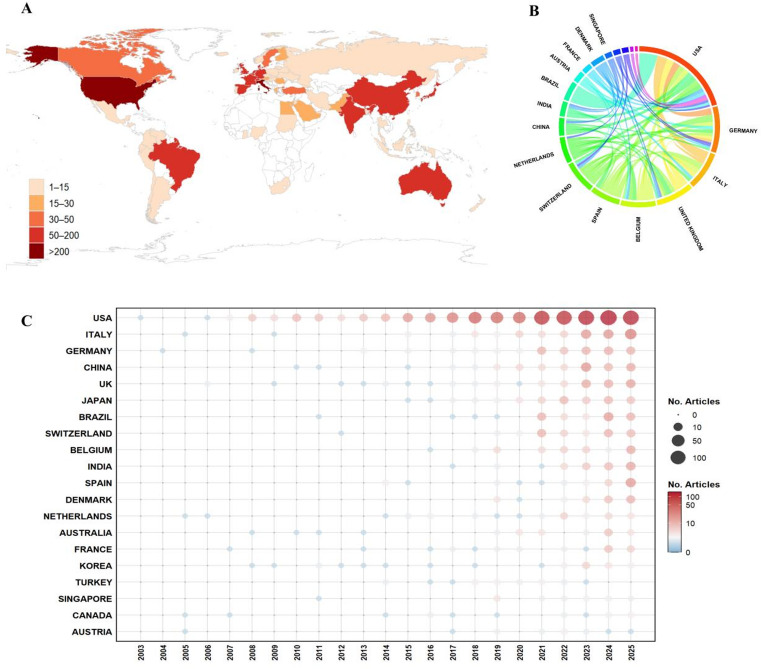
Productivity and Collaboration Among Nations in Robotic Hernia Repair Research.** A** Geographical distribution of the number of national publications. **B** Chord diagram of international collaborations among countries. **C** The annual number of papers published by the top 20 countries

There are notable differences in the developmental timelines among these leading countries. As illustrated in Fig. 2C, the United States was the earliest pioneer to initiate research in 2003 and maintains the longest continuous research span. Italy and Germany followed, gradually accelerating their relevant research efforts around 2011. China entered the field in 2015 but has rapidly expanded its output to secure the fourth position globally with 68 articles (4.52%). Since the historic turning point in 2020, nearly all top producing countries have consistently published research annually. Geographically, as depicted in the world map in Fig. 2A, nations in North America and Europe have extensively participated in this domain, with their output significantly surpassing that of other regions. In contrast, countries on the African continent have exhibited minimal involvement in this technological evolution.

The United States demonstrates the highest frequency of international partnerships, collaborating with 60 different nations. European countries also show robust cooperative network, with Italy and Germany engaging 43 and 39 partner countries respectively. Conversely, certain Asian and South American nations exhibit more localized research dynamics. For instance, Japan and Brazil maintain fewer international collaborations, connecting with only 14 and 19 partner countries respectively. These intricate international cooperative networks are visually summarized in the chord diagram in Fig. 2B, which highlights the central node position of the United States and the dense collaborative ties within Europe.

In addition, given the dominant role of the United States and the rapid rise of China in the global output of RHR research, a descriptive comparison of these two research systems is provided in Supplementary Fig. 3.

**Table 1:** Basic Information of the Top 10 Most Productive Countries in Robotic Hernia Repair Research

Rank	Country	Output (*N* = 1503, n%)	Citations (*N* = 19258, n%)	Partner countries (*N* = 72, n%)	2025GDP Rank	United Nations region
1	United States	892 (59.35)	12,914 (67.06)	60 (83.33)	1	Americas
2	Italy	98 (6.52)	1687 (8.76)	43 (59.72)	8	Europe
3	Germany	71 (4.72)	1384 (7.19)	39 (54.17)	3	Europe
4	China	68 (4.52)	949 (4.93)	33 (45.83)	2	Asia
5	United Kingdom	66 (4.39)	1359 (7.06)	38 (52.78)	6	Europe
6	Japan	54 (3.59)	330 (1.71)	14 (19.44)	4	Asia
7	Brazil	52 (3.46)	319 (1.66)	19 (26.39)	11	Americas
8	Switzerland	47 (3.13)	906 (4.70)	22 (30.56)	21	Europe
9	Belgium	40 (2.66)	1006 (5.22)	26 (36.11)	23	Europe
10	India	39 (2.59)	640 (3.32)	33 (45.83)	5	Asia

### Author analysis

**Fig. 3:**
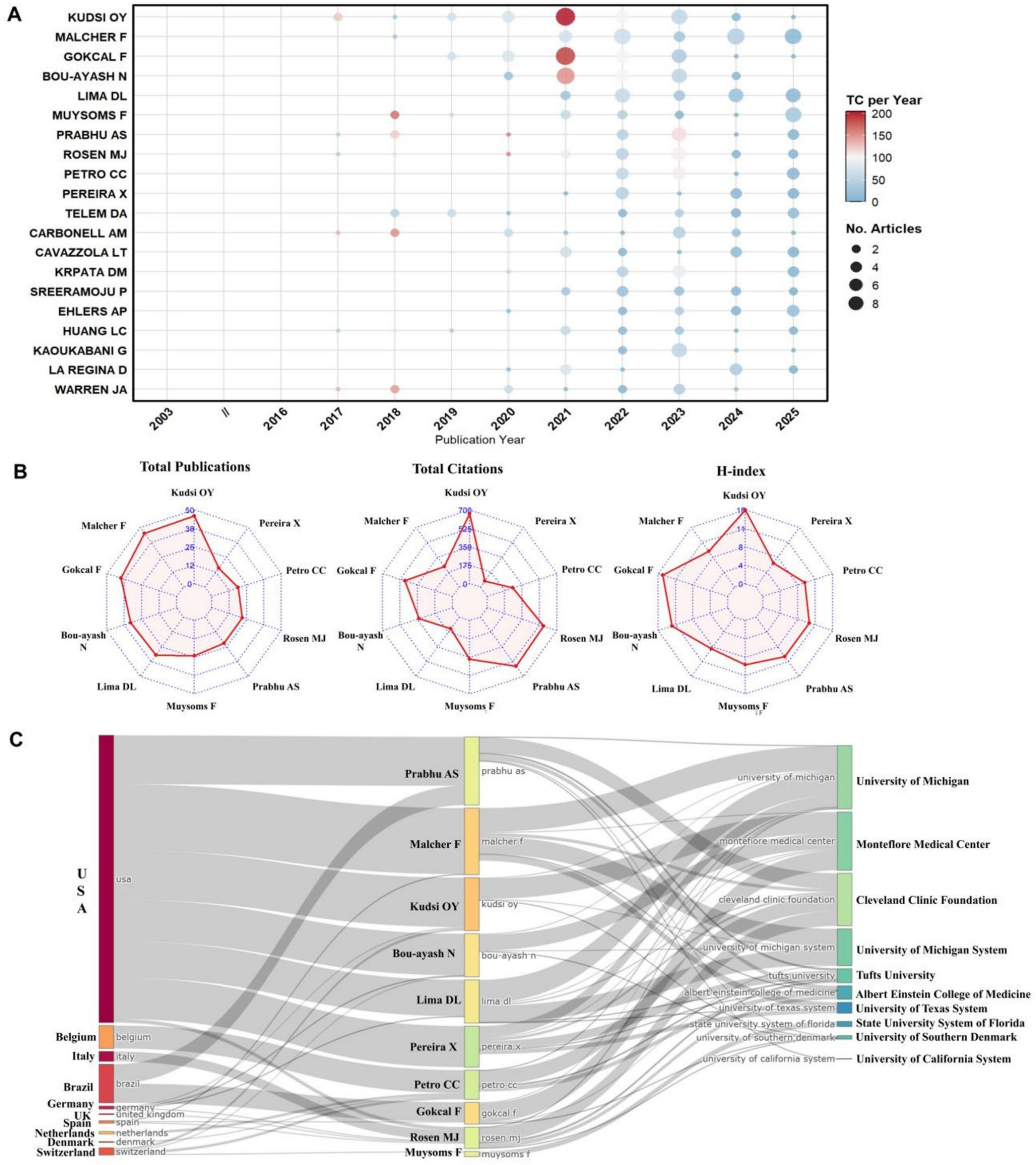
Author correlation analysis. **A** The annual number of papers published by the top 10 authors and the average annual number of citations. **B** Total Citation and H-index of authors with the top 10 publications. **C** The author–countries– institutes Sankey diagram

Based on the macroscopic overview of national publication trends, we further characterized the key individual contributors and the underlying structure of collaboration driving research in RHR. As visualized through the multidimensional radar metrics in Fig. 3B, Kudsi OY unequivocally dominates the field, leading simultaneously in total publications, total citations, and H index. Other highly productive and impactful investigators include Malcher F, Gokcal F, Bou Ayash N, and Muysoms F. The robust performance across all three metrics indicates that their high productivity is deeply coupled with sustained scholarly influence. The annual publication trajectories presented in Fig. 3A further reveal that while early pioneers like Muysoms F established the foundational literature, authors such as Kudsi OY and Malcher F have experienced explosive output growth since the 2020 turning point.

Moving beyond absolute publication counts, the topology of the collaboration networks reveals important features regarding how this research is organized. As detailed in Table 2, a small group of researchers occupies the central positions. In the concurrent citation network Muysoms F and Kudsi OY exhibit the highest centrality scores of 0.53 and 0.18 respectively, indicating their foundational status in the intellectual consensus of the field. Within the active collaboration network, Malcher F, Rosen MJ and Morales-conde S emerge as the most central nodes. These pivotal individuals link otherwise isolated research groups, facilitating the knowledge flow and methodological standardization across different institutions.

The Sankey diagram in Fig. 3C emphasizes the deep institutional roots supporting these global collaboration. The United States maintains a commanding presence in this area, acting as the central base for numerous top researchers like Kudsi OY, Malcher F and prabhu AS. These researchers have forged strong ties with esteemed academic institutions, notably the University of Michigan, Montefiore Medical Center and the Cleveland Clinic Foundation. Conversely, Muysoms F anchors a vital European network based in Belgium. In summary, the current landscape is characterized by a few highly influential and deeply interconnected hubs largely concentrated in the United States, highlighting an urgent necessity to broaden cross-regional partnerships to sustain and accelerate global scientific advancement.

### Journal analysis

**Fig. 4:**
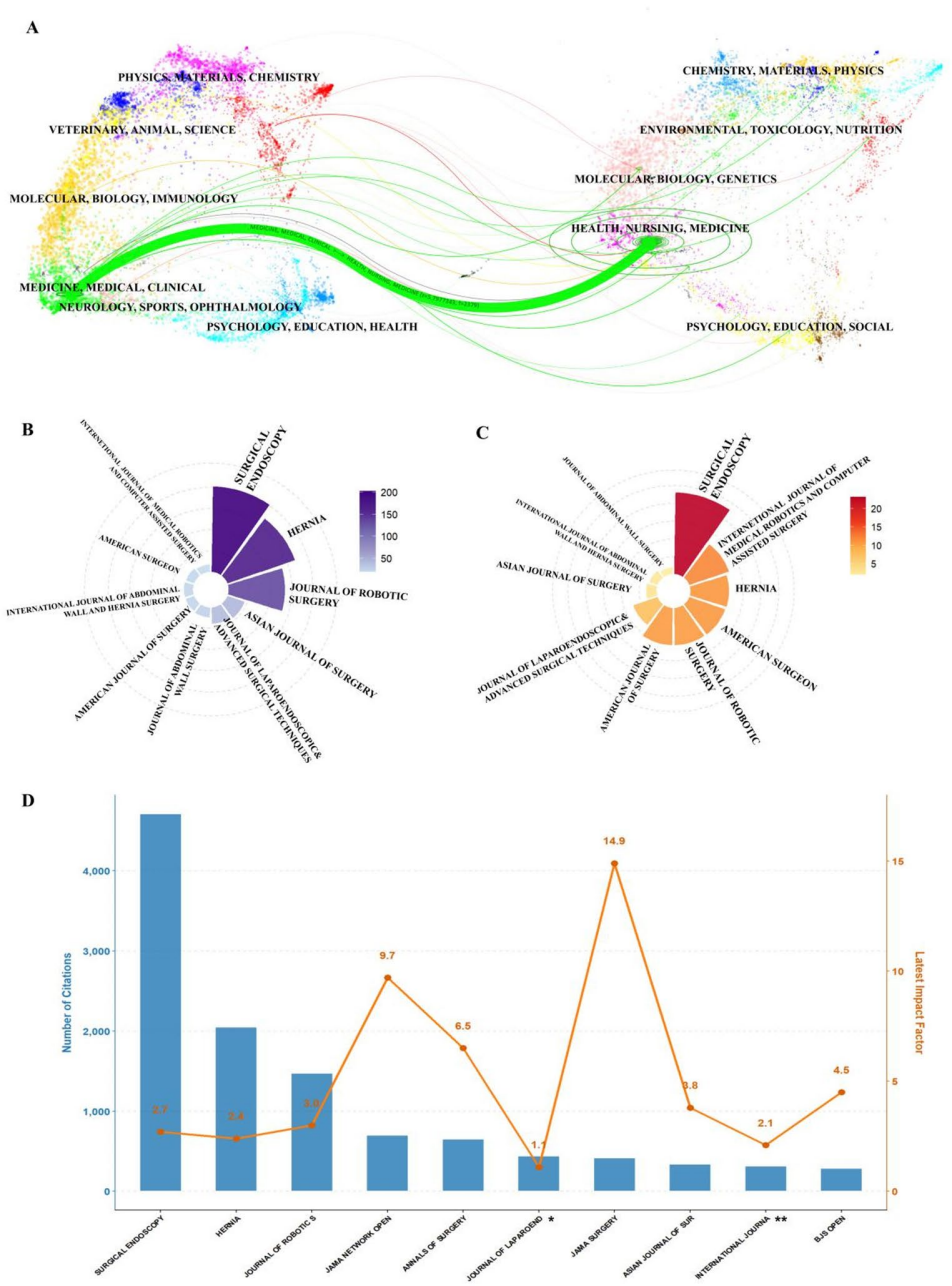
Correlation analysis of journals.** A** The double graph superposition of the citation domain and the cited domain of the journal. **B** The average number of citations of the top 10 published journals. **C** The average number of publications of the top 10 published journals. **D** The number of citations of the top 10 journals and the impact factor in 2025. (* JOURNAL OF LAPAROENDOSCOPIC & ADVANCED SURGICAL TECHNIQUES, ** INTERNATIONAL JOURNAL OF MEDICAL ROBOTICS AND COMPUTER ASSISTED SURGERY)

Building upon the major authors and collaboration patterns, we subsequently evaluated the core academic journals disseminating research on RHR. An examination of dual map overlay in Fig. 4A indicates the fundamental knowledge flow and citation trajectories. The research predominantly originates from journal groups of medicine, medical, clinical, and is mainly cited by health, nursing, medicine. The citation domain on the left side has an outward main citation path (MEDICINE, MEDICAL, CLINICAL → HEALTH, NURSING, MEDICINE), which is the most significant citation group with a Z value of 5.798. This high Z value indicates a statistically prominent citation flow. Substantively, this pathway demonstrates that RHR research is anchored in broader nursing and healthcare literature rather than isolated surgical technicalities, which aligns with the transition toward value-based care.

Regarding specific publication volumes detailed in Table 3 and visualized through the radial charts in Fig. 4B and C, SURGICAL ENDOSCOPY emerges as the most prolific venue, contributing 203 articles which account for 14% of the total global output. The journal HERNIA ranks second with 170 publications representing 11%, followed by the JOURNAL OF ROBOTIC SURGERY with 135 articles at 9%. These 4 journals alone consolidate a massive portion of the core research, serving as the primary academic forums for technical exchange in domain.

When assessing broader academic influence through Fig. 4D, SURGICAL ENDOSCOPY unequivocally commands the highest total citation count exceeding 4000 citations due to its historical accumulation and high output volume. Specifically, JAMA SURGERY leads the entire cohort with an exceptional impact factor of 14.9. JAMA NETWORK OPEN and ANNALS OF SURGERY follow closely with impact factors of 9.7 and 6.5 respectively. Therefore, this illustrates that the research on RHR has been generally recognized by high quality journals.

**Table 2:** Co-authors and Co-cited Authors with Co-occurrence Network C entrality

Rank	Cited author	Counts	Cited author	Centrality	Author	Centrality
1	Muysoms F	305	Muysoms F	0.53	Malcher F	0.04
2	Kudsi OY	297	Kudsi OY	0.18	Rosen MJ	0.03
3	Bittner R	174	Dindo D	0.13	Morales-conde S	0.03
4	Köckerling F	167	Simons MP	0.10	Lee B	0.03
5	Belyansky I	164	Prabhu AS	0.09	Lima DL	0.02
6	Simons MP	150	Köckerling F	0.08	Prabhu AS	0.02
7	Petro CC	144	Bittner R	0.07	Petro CC	0.02
8	Prabhu AS	140	Poulose BK	0.07	Cavazzola LT	0.02
9	Bittner JG	111	Belyansky I	0.06	Huang LC	0.02
10	Aiolfi A	110	Finley DS	0.05	Dietz UA	0.02

### Citations and references analysis

**Fig. 5:**
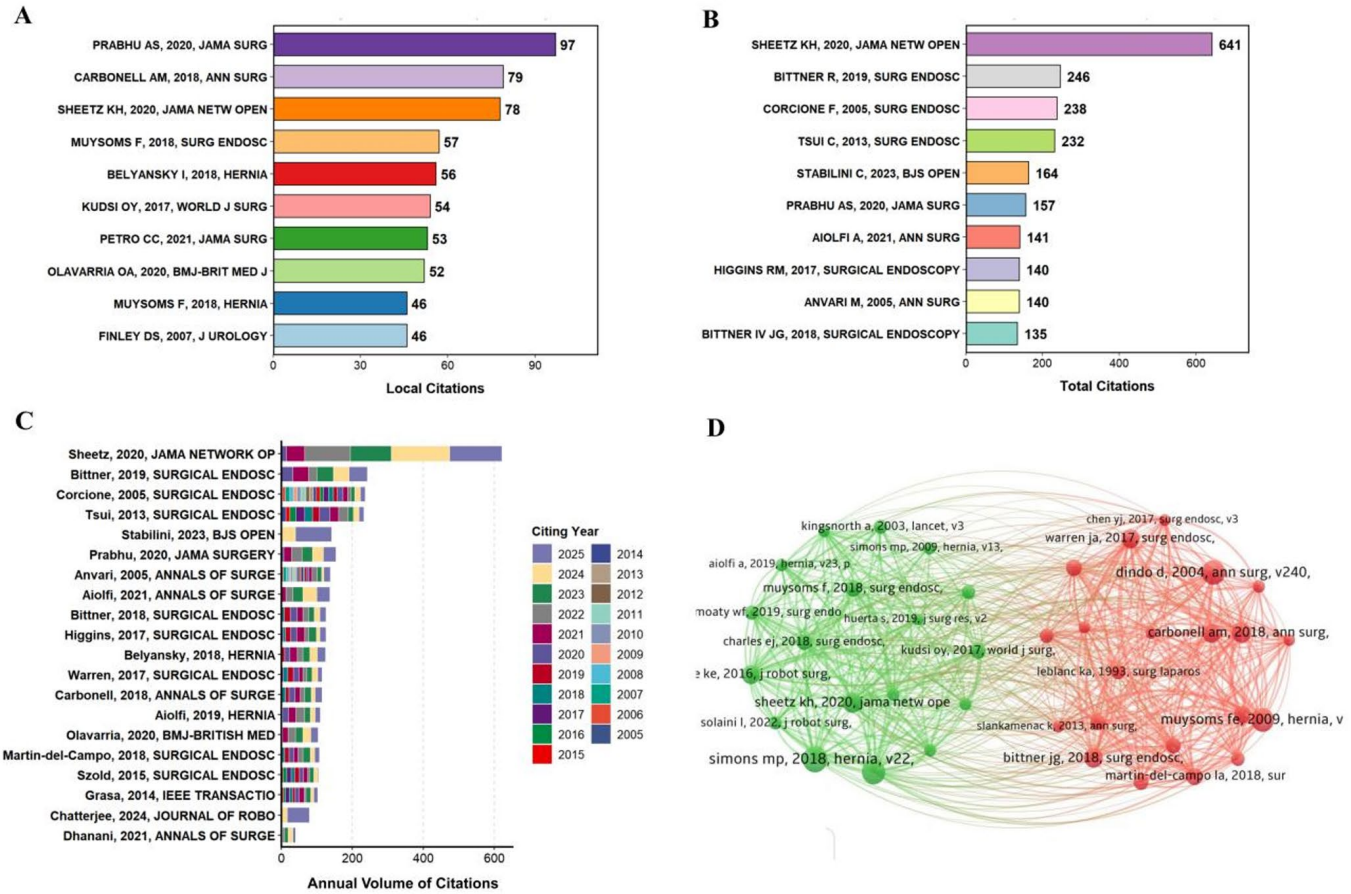
Important literature and references analysis.** A** Top 10 citations in local literature. **B** Top 10 total citations. **C** Annual literature citations of the top 20 total citations. **D** Coupling analysis of the citation literature

To gain insight into the foundational knowledge and intellectual structure of RHR, we conducted an evaluation of the most influential research. By examining the overarching influence detailed in Fig. 5B, the research conducted by Sheetz KH in 2020 unequivocally dominates the global landscape. This landmark paper has accumulated 641 total citations and 78 local citations. Furthermore, Fig. 5C illustrates a sustained rise in annual citations in recent years, highlighting its enduring importance in contemporary research on surgical outcomes. Prabhu AS also made a major contribution to development of RHR. As depicted in Fig. 5A, the research authored by Prabhu AS secured the highest local citation count of 97 in our dataset while also accumulating 157 total citations. It indicates that this research has had strong influence within the specialized research on RHR. Additionally, the comprehensive research conducted by Bittner R in 2019 and older seminal publications such as the research by Corcione F in 2005 maintain robust total citations counts of 246 and 238 respectively. The longitudinal trends Fig. 5C reveals a evolutionary dynamic. With most of research published around 2020 exhibit an upward trajectory in their annual citation, whereas the foundational publications from earlier years have maintained stable and sustained scholarly influence.

To map the underlying conceptual relationships among these high-cited research, we performed a bibliographic coupling analysis. As shown in Fig. 5D, the network demonstrates intense interconnections and forms two major thematic clusters. The green cluster was largely anchored by the research of Muysoms F, Kudsi OY and Simons MP. In contrast, the adjacent red cluster was mainly centered on Carbonell AM and Dindo D.

### Analysis of institutions contribution

**Fig. 6:**
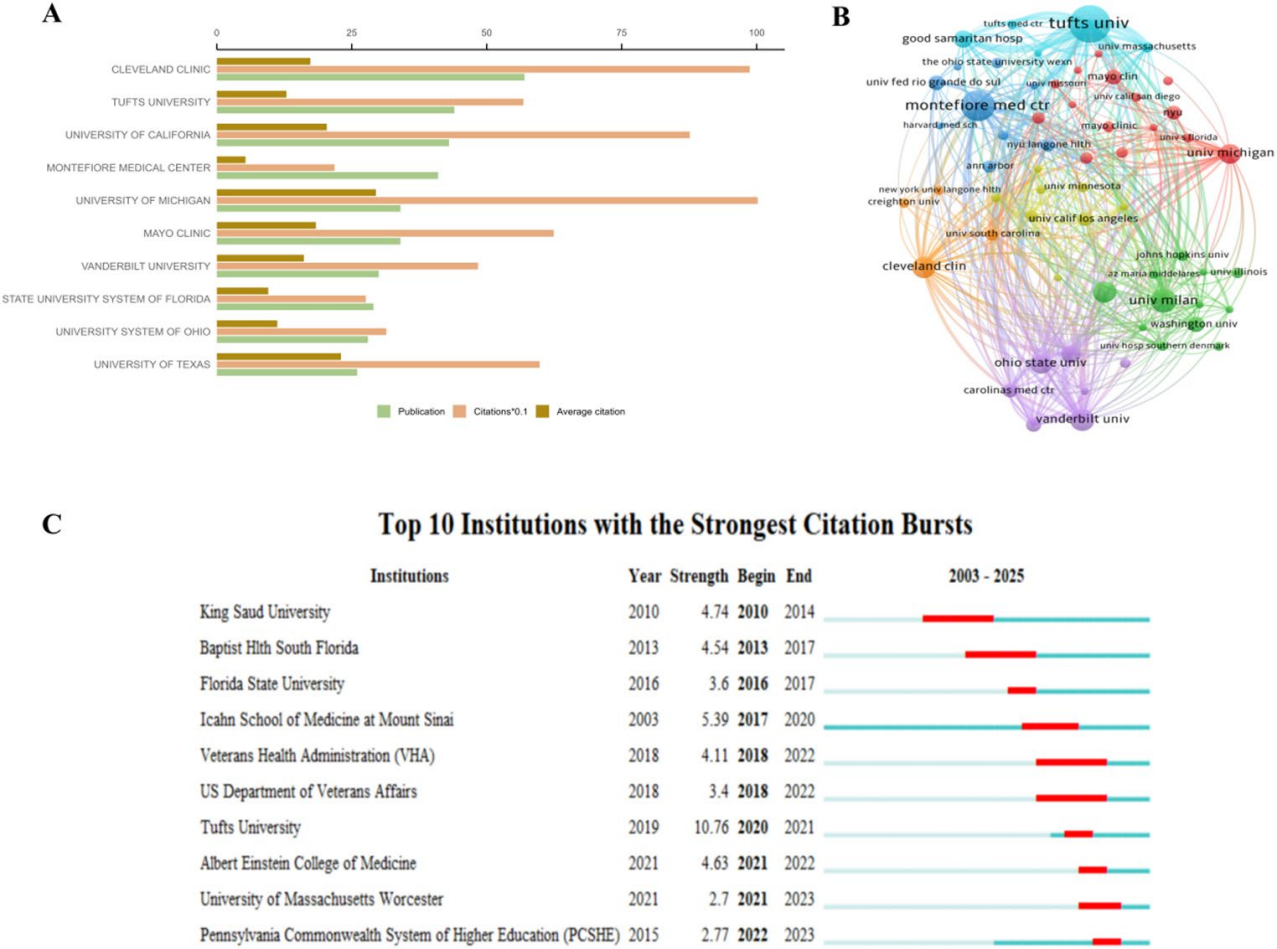
Analysis of related institution.** A** The number of publications and citations of the institutions of the co-authors. **B** Cooperation between the countries of the co-authors. **C** The institutions of the top 10 strongest citation bursts

To understand the organizational framework driving RHR research, we conducted a systematic institution analysis. Academic influence within the field of RHR is heavily consolidated among several elite centers. As shown in Fig. 6A, the University of Michigan establishes itself as the foundational pillar, ranking first globally in both absolute publication volume and cumulative citations. Notably, Fudan University exhibits a uniquely high average citation rate despite a comparatively modest publication output, indicating profound individual article impact rather than volumetric dominance.

Beyond isolated productivity, spatial institutional networks illustrate a tight collaborative topography (Fig. 6B). Within this complex structure, the Tufts University emerges as a pivotal hub. As demonstrated by its high node centrality and dense connections, the Cleveland Clinic plays a leading role in inter-institutional collaboration, effectively linking various academic clusters alongside other core institutions such as the University of Michigan and Montefiore Medical Center.

To map the timeline of academic leadership, CiteSpace burst analysis was used to identify institutions with sudden increases in citations (Fig. 6C). The historical trend reveals a constantly shifting leadership landscape. Although early growth was led by King Saud University (strength: 4.74, 2010–2014), the most significant changes occurred recently. Notably, Tufts University showed a significantly strong citation burst (strength: 10.76) from 2020 to 2021. Together with subsequent bursts from the Albert Einstein College of Medicine and Pennsylvania Commonwealth System of Higher education, this indicates a rapid and continuous shift of academic focus toward newly established clinical centers.

### Keyword analysis

**Fig. 7:**
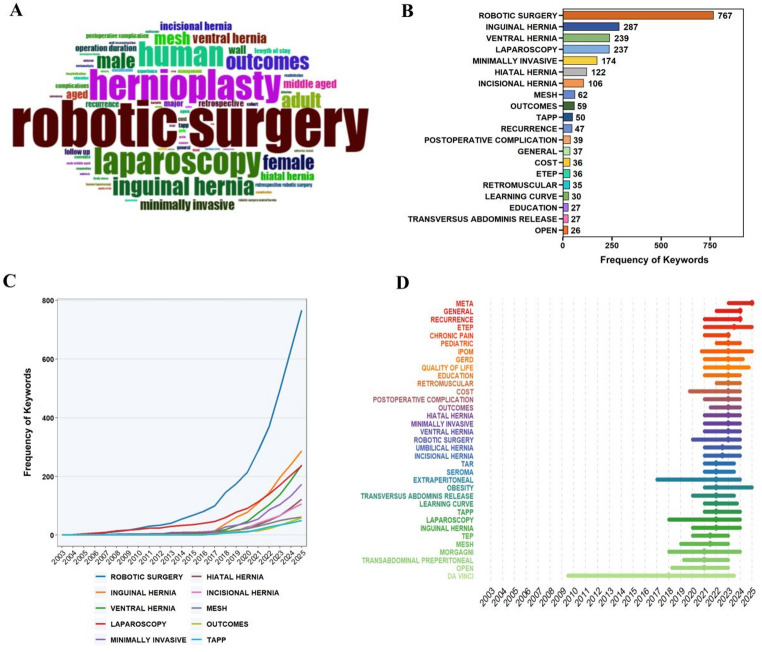
Keyword analysis.** A** Word cloud of keywords. **B** Frequency distribution of top 20 keywords. **C** Growth dynamic of top 10 keywords. **D** Timeline of keywords occurrences

To elucidate the thematic evolution and emerging research focuses in the field of RHR, we conducted a keyword analysis. As illustrated in Fig. 7A, the keyword cloud indicates that the predominant research directions center around robotic surgery, hernioplasty, laparoscopy and specific hernia types. The frequency distribution (Fig. 7B) highlights that “robotic surgery” (767) is the absolute core keyword, followed by “inguinal hernia” (287), “ventral hernia” (239), “laparoscopy” (237), and “minimally invasive” (174).

The growth dynamic of high-frequency keyword (Fig. 7C) demonstrates that the use of these keywords has increased rapidly in recent years. Notably, the term “robotic surgery” exhibits a surge beginning around 2017, which significantly surpasses other keywords. This indicates a paradigm shift toward the adoption of robot-assisted techniques in hernia repair.

As shown in Fig. 7D, we also analyzed the timeline of keyword occurrences to distinguish between established concepts and emerging clinical trends. Traditional concepts such as “ Da Vinci”, “open” display sustained relevance over an extended period, which reflects that they serve as the backbone of RHR. In contrast, keywords such as “ETEP”, “quality of life”, “cost” and “education” have emerged prominently within the last three to five years. This temporal shift suggests that the researchers not only explore novel techniques, but also prioritize outcomes, healthcare economics and the learning curves associated with surgical training.

**Table 3:** The Publications of the Top 10 Journals

Journal	Publications	Percentage
SURGICAL ENDOSCOPY	203	14%
HERNIA	170	11%
JOURNAL OF ROBOTIC SURGERY	135	9%
ASIAN JOURNAL OF SURGERY	45	3%
JOURNAL OF LAPAROENDOSCOPIC & ADVANCED SURGICAL TECHNIQUES	41	3%
JOURNAL OF ABDOMINAL WALL SURGERY	26	2%
AMERICAN JOURNAL OF SURGERY	24	2%
INTERNATIONAL JOURNAL OF ABDOMINAL WALL AND HERNIA SURGERY	24	2%
AMERICAN SURGEON	23	2%
INTERNATIONAL JOURNAL OF MEDICAL ROBOTICS AND COMPUTER ASSISTED SURGERY	20	1%

### LDA-based topic modeling

We applied LDA to the corpus to yield 8 distinct thematic domains. Topic labels for each topic were assigned through manual review of the top 10 terms. As shown in Table 4, prominent topics included “Topic 2: Complex atypical hernia repair” (*n* = 273; 0.18) and “Topic 1: Advanced abdominal wall reconstruction” (*n* = 219; 0.15), followed by “Topic 8: Health economic evaluation” (*n* = 203; 0.14), “Topic 7: High-risk obese demographic management” (*n* = 197; 0.13), and “Topic 5: Routine inguinal hernia repair” (*n* = 182; 0.12).

**Fig. 8:**
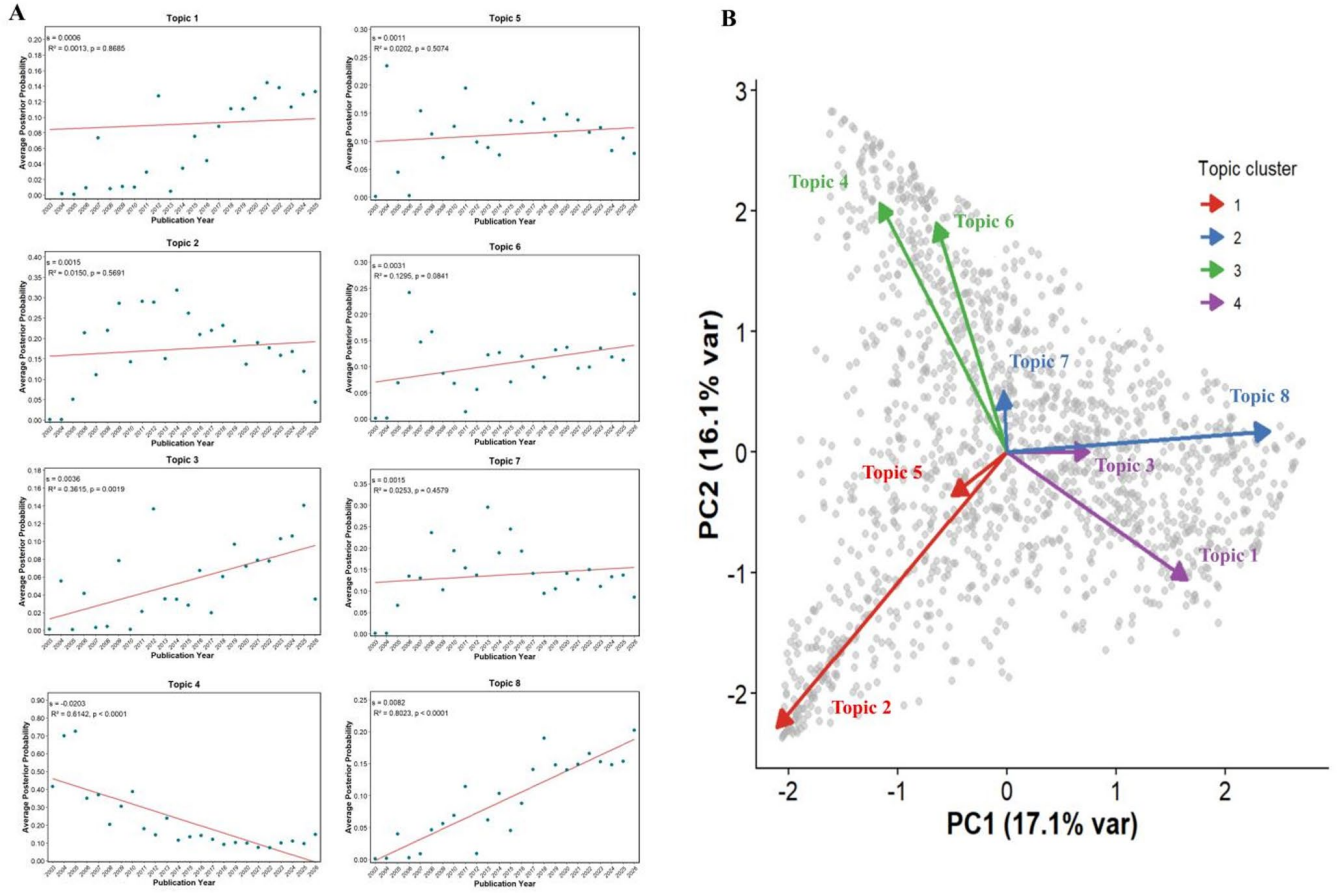
Topic Dynamics and Document–Topic Structure:** A** Temporal trends of latent topics on RHR (2003–2025). **B** Husson–Jongmans biplot of the document–topic distribution

To trace the evolutionary trajectory of these themes, longitudinal linear regression analysis was performed. Publication year served as the independent variable with the average posterior topic probability acting as the dependent variable. Trend were classified based on the slope and statistical significance (threshold: *P* < 0.01). As detailed in Fig. 8A, Topic 8 (Health economic evaluation; *P* < 0.001) and Topic 3 (Evidence based prognostic evaluation; *P* = 0.0019) demonstrated significant growth. Conversely, Topic 4 (Robotic platform evolution, *P* < 0.0001) experienced a decline, while the remaining topics exhibited no significant temporal change (*P* > 0.01), suggesting relative stability.

We applied principal component analysis to the document-topic distribution matrix (θ). In the resulting biplot (Fig. 8B), the first two principal components explained 17.1% (PC1) and 16.1% (PC2) of the variance. The gray points represent individual documents and colored arrows denotes topic vectors. Longer arrows indicate stronger contributions to the component, with documents located in the direction of an arrow showing higher posterior probabilities for that corresponding topic. In this reduced two-dimensional space, the eight topic vectors were visually grouped into four major clusters: Cluster 1 (Red) delineates the broad “Anatomical Spectrum”, bridging standard procedures (Topic 5) and highly demanding atypical repairs (Topic 2). Cluster 2 (Blue) addresses resource management, linking the surgical challenges of obese demographics (Topic 7) directly with health economics (Topic 8). Cluster 3 (Green) reflects the critical interface between hardware and humans, grouping the adoption of novel robotic systems (Topic 4) with the learning curves of surgical training (Topic 6). Cluster 4 (Purple) demonstrates a relationship between technical optimization and clinical validation, coupling highly complex abdominal wall reconstructions (Topic 1) with robust evidence based prognostic evaluations (Topic 3).

**Table 4:** Topic Discovered from Articles From 2003 to 2025

Topic	Prevalence	Top terms	Label	*N*
1	0.15	mesh, ventral hernia, robotic surgery, incisional hernia, outcomes, ETEP, wall, TAR, defect, cm	Advanced abdominal wall reconstruction	219
2	0.18	robotic surgery, mesh, laparoscopy, approach, invasive, minimally, parastomal hernia, rare, bowel, diaphragmatic	Complex atypical hernia repair	273
3	0.09	outcomes, laparoscopy, pain, quality, systematic, robotic surgery, life, recurrence, meta, postoperative	Evidence based prognostic evaluation	131
4	0.10	robotic surgery, laparoscopy, system, outcomes, single, RAS, Da Vinci, minimally, platform	Robotic platform evolution	144
5	0.12	inguinal hernia, robotic surgery, TAPP, laparoscopy, preperitoneal, outcomes, inguinal, transabdominal, TEP, mesh	Routine inguinal hernia repair	182
6	0.10	robotic surgery, surgeons, invasive, minimally, resident, training, based, surgeon, outcomes, learning	Standardized surgical training	150
7	0.13	hiatal hernia, outcomes, robotic surgery, complications, median, BMI, age, follow, months, kg	High-risk obese demographic management	197
8	0.14	robotic surgery, outcomes, laparoscopy, cost, compared, ventral hernia, approach, versus, risk, day	Health economic evaluation	203

## Discussion

### Temporal dynamics

Longitudinal time-series analysis of global publication output revealed a developmental trajectory punctuated by two statistically significant time points. 2016 and 2020 were identified by joinpoint regression, partitioning the evolution of RHR into three distinct stages. The initial latency period (2003–2015) represented merely 8.38% of the total publications, which reflected the hardware constraints of early-generation robotic platforms. The bulky mechanical arms and complicated docking procedures made the robotic platforms cumbersome in complex hernia repair. Therefore, robotic surgery during this stage was largely confined to pelvic procedures, while its application in hernia repair was limited to single institution feasibility studies [24–26]. The 2016 inflection point marked a slope escalation from 1.53 to 14.00, coinciding with the widespread adoption of the da Vinci platform whose suspended rotatable arms resolved the limitations of instrument collision and enabled the systematic codification of r-TAR and r-eTEP [9, 27]. The most dramatic acceleration emerged since 2020, when publications constituted 71.86% with a slope of 36.11. We speculated that this acceleration was temporally associated with the convergence of several landmark publications, including the RIVAL trial and large scale analyses from the Michigan Surgical Quality Collaborative [5, 28, 29]. Simultaneously, the COVID-19 pandemic compelled surgeons to prioritize strategies that minimized hospital length of stay and interpersonal contact [30, 31]. Robotic platform demonstrated distinctive advantages in this context. The pandemic not only accelerated the popularization of RHR, but generated a substantial academic output.

### Geospatial distribution

Analysis of national productivity and collaboration networks revealed a unequal distribution of knowledge production of RHR. The top 10 most productive countries accounted for 94.93% of total publications. This pattern reflects not only differences in research capacity, but also the influence national healthcare financing systems on surgical technology adoption.

The United States established dominance in publication volume (892), citations (12914) and collaboration centrality. This output is closely tied to the structure of the American healthcare market. The hospitals frequently adopt robotic technology as a competitive tool to attract commercially insured patients and recruit skillful surgeons [28, 32]. Research suggests that patients in highly competitive markets are 2 to 5 times more likely to undergo robotic procedures than those in less competitive settings [33]. This phenomenon has driven the massive clinical practice and academic output.

This market-driven pattern contrasts with the adoption logic of publicly funded healthcare systems. In these settings, robotic surgery is often evaluated through health technology assessment (HTA) frameworks which consider comparative effectiveness, cost-effectiveness, budget impact, organizational requirements and evidence generation. HTA guidance from agencies such as NICE, HAS and NIPH provides an important context for understanding the more cautious and value-oriented diffusion of robotic surgery in European countries [34–36].

European countries including Italy, Germany and the United Kingdom present a contrasting phenomenon. Constrained by publicly funded healthcare systems and strict medical budget, European researchers approached RHR with scrutiny [37, 38]. Their research focused more on cost-effectiveness analysis and evidence based guideline development [37, 39]. Therefore, the European Hernia Society has been active in producing guideline iterations designed to prevent robotic adoption in cases where routine operations offer comparable outcomes.

At the author level, American researchers including Kudsi OY and Malcher F led in productivity and h-index values, while European researchers Muysoms F achieved the highest co-citation centrality at 0.53. This suggests that American researchers prioritized high academic output and European researchers focused on conceptual framework building. Institution mapping further revealed that core research remained concentrated within elite centers such as the University of Michigan and the Cleveland Clinic, indicating the need to extend collaborative networks to lower resource settings in developing countries, where the applicability of RHR under different economic conditions remains largely unexplored.

### Knowledge flows and evidentiary disruption

Dual-map overlay analysis illuminated that citation trajectories in RHR flowed from “Medicine, Medical, Clinical” toward “Health, Nursing, Medicine” with a high pathway strength of Z = 5.798. This pattern reflects a fundamental shift from technical execution to perioperative care quality, patient recovery trajectories and long-term quality of life assessment. The strong citation performance of high-impact journals including JAMA Surgery and Annals of Surgery confirms that RHR has moved beyond its origin as a niche technical subspecialty and entered the mainstream of surgical academic discourse.

However, the recognition inevitably attracted greater academic scrutiny. The pressure is most clearly illustrated by two landmark publications. The most globally cited literature which was published by Sheetz et al. in JAMA Network Open in 2020 (641) indicated that there is an 8.4-fold increase in robotic platform across routine American surgical procedures within 6 years [28]. This rise was particularly striking in inguinal hernia repair, which climbed from 0.7% to 28.8%, representing a 41.1-fold increase. Seriously, this expansion occurred alongside a decline in conventional laparoscopic use, raising concerns regarding potential overuse of robotic technology in the absence of clinical benefit. The RIVAL trial which received the highest local citation density responded to this concern [5]. This trial was the first multicenter randomized pilot trial comparing robotic versus laparoscopic TAPP inguinal hernia repair. It demonstrated no significant differences in postoperative pain, quality of life or wound outcomes for unilateral hernia, while revealing longer operative time, substantially higher costs and paradoxically increased surgeon frustration in the robotic group. These two publications prompted critical reassessment of the role of RHR. In the absence of clear clinical or ergonomic advantages in routine cases, the field began to redefine its appropriate scope of application. Keyword timeline analysis confirms this reorientation, which shows the decline of broad term such as “da Vinci” and “laparoscopy” alongside the sharp emergence of “eTEP”, “TAR”, “quality of life”, “cost” and “education”. This field has shifted from mechanical validation towards complex reconstruction and multidimensional value assessment [9, 39].

### Four major research domains in RHR

This study applied PCA to the LDA-derived topic matrix and constructed a HJ-Biplot to visualize the relationships among the eight latent topics. The topic vectors formed four visually recognizable clusters in the reduced two-dimensional space. Given the first two principal components explained a moderate proportion of the total variance, these clusters were mainly used as an exploratory visualization. Therefore, we summarized the thematic structure of RHR into four major research domains.

#### Domain one: The anatomical spectrum (Red cluster)

The co-localization of routine inguinal repair (Topic 5) and rare complex hernias (Topic 2) reflects the full procedural spectrum of RHR. Routine inguinal repair, despite ongoing concerns regarding to cost-effectiveness, remains the most common entry point for surgeons to build robotic experience due to its low procedural risk [5, 40]. Topic 2 covers cases including parastomal hernias and deep pelvic floor defects, where conventional laparoscopy is limited by restricted visibility and instrument maneuverability. In these settings, robotic platforms offer genuine technical advantages that facilitates precise dissection within confined anatomical spaces [9, 41, 42]. These findings indicate that high-volume routine procedures serve to establish the surgical confidence and proficiency of platforms, which provide the foundation for extending robotic application into complex cases.

#### Domain two: The balance between resource and risk management (Blue cluster)

The pairing of high-risk populations such as morbid obesity and giant hiatal hernia (Topic 7) with health economics (Topic 8) reflects a clinical-economic coupling. Patients with BMI exceeding 40 undergoing complex abdominal wall reconstruction or hiatal repair consume more operative resources and face significantly higher rates of pulmonary infections and wound infection [43]. Given the basic cost of robotic platforms at approximately $3000 to $5000 per cases, any additional complication worsens the financial equation [14]. Consequently, research within this cluster has increasingly focused on whether robotic approaches can compensate these costs by reducing length of stay and complication rates, thereby enabling selected high-risk cases to be managed within accelerated recovery or even ambulatory pathways [44]. If achieved, the downstream savings from reduced bed occupancy and complication management may compensate for the technological costs.

#### Domain three: Hardware evolution and surgical education (Green cluster)

The green cluster pairs robotic platform development (Topic 4) with surgical education and learning curve (Topic 6). While the accumulated bibliometric data reflects the historical dominance of da Vinci multi-arm systems, recent platform development is rapidly diversifying across two distinct axes: manufacturer competition and architectural innovation. Regarding manufacturer competition, newer competitive systems (Versius, Hugo RAS and Senhance) and emerging domestic Chinese platforms (KangDuo and MicroPort Toumai) are actively challenging this monopoly with novel modular and open-console designs. Regarding architectural innovation, the da Vinci Single port (SP) system introduces a different access paradigm [45, 46]. Unlike conventional multi-arm systems, the SP architecture utilizes a single-incision model. This hardware innovation creates a ripple effect across all thematic domains. Anatomically, SP applications have been extended to extraperitoneal approaches (r-eTEP SP, SP^2^ eTEP), which minimize abdominal wall trauma [47, 48]. Clinically and economically, single-port approaches may offer meaningful reductions in access-related morbidity which create a distinct health-economic profile [49]. Furthermore, the SP system requires a steeper learning curve and restructured training pathways [50]. Recent literature demonstrates that both these alternative platforms and the novel SP architecture offer feasible clinical outcomes. Consequently, the future direction for this field is the transition from single-platform feasibility reports toward rigorous multi-platform comparative effectiveness studies [51–53].

As the hardware landscape becomes increasingly diversified, each iteration upgrade in robotic platform demands a corresponding restructuring of how surgeons are trained [54]. Unlike open or laparoscopic surgery where the mentor and trainee operate side by side, robotic surgery separates the operating surgeon from the sterile field through its console. This alters the traditional mentor-trainee interaction and increases the need for simulation, dual-console supervision and objective feedback [55, 56]. Simulation-based curricula like the Fundamental Skills of Robotic Surgery program have been validated for basic robotic skill acquisition, while proficiency-based progression emphasizes advancement after predefined performance benchmarks are achieved rather than after a fixed number of operations [56, 57]. Objective assessment tools including OSATS-derived robotic assessments (R-OSATS and GEARS) further provide structured methods to evaluate robotic technical skills [58, 59]. In abdominal wall surgery, the European Hernia Society has described a staged training pathway for robotic abdominal wall surgery, including platform familiarization, preclinical training, supervised clinical introduction and procedure-specific mentoring [56]. Procedure-specific studies using CUSUM analysis in robotic TAPP inguinal hernia repair also show that operative performance improves with experience, reinforcing the need for structured assessment during the learning curve [60]. Therefore, this domain reflects a broader shift from informal apprenticeship toward simulation-supported and objectively assessed robotic surgical education [61, 62].

#### Domain four: Technical complexity and high-level evidence (Purple cluster)

The purple cluster pairs advanced abdominal wall reconstruction including rTAR and r-eTEP (Topic 1) with high-level evidence and pain management outcomes (Topic 3). These procedures represent one of the most technically demanding frontiers of RHR [63, 64]. They require extensive dissection through avascular planes between abdominal wall muscle layers to place mesh in the retromuscular space, which avoids the drawbacks of intraperitoneal mesh contact with the viscera [65]. Because these procedures involve extensive dissection fields and the complex anatomy of layers, they have generated increasing demand for rigorous evidence. Therefore, recent research has shifted toward systematic reviews and RCT, which aimed at defining the clinical value of robotic platforms in these complex procedures.

### Three thematic transitions in RHR

Longitudinal linear regression of LDA topic posterior probabilities revealed three dynamic thematic transitions which mark the transition of RHR from an stage of unconstrained growth into a disciplined and evidence-based stage.

#### Thematic transition one: the awakening of value-based healthcare

The temporal evolution of the literature demonstrates a critical thematic shift, which is highlighted by the significant growth of Topic 8. This trend indicates that the current research priorities have evolved from technical optimization to rigorous economic evaluation. Driven by the sustainability challenges of global healthcare systems, researchers are undertaking cost-benefit analysis. Recent studies report an incremental cost-effectiveness ratio of €1,149 per hospital day saved by robotics platforms [44]. Such analysis demonstrates that minor short-term pain reduction cannot compensate the expensive robotic consumables (About $3000-$5000 incremental cost) [14]. However, a clear economic crossover point emerges in this context. This value is realized when robotic platforms prevent incisional infections in morbidly obese patients with wide defects. Consequently, this transition establishes “value-based healthcare” as the ultimate guiding principle for future robotic applications.

#### Thematic transition two: from technical prowess to rigorous evidence-based scrutiny

The second major development in the longitudinal trend analysis was the significant rise of Topic 3, which reflects high-level evidence and prognostic evaluation. In contrast, Topic 1, which focused on advanced reconstructive techniques remained in a stage of steady accumulation. From 2017 to 2020, research was primarily driven by the technical optimization of r-eTEP and rTAR procedures [63, 65]. Nevertheless, publication of RCTs such as RIVAL trials showed that robotic surgery offers no significant advantages over laparoscopy in certain type of hernias. This evidence promoted a reappraisal of RHR [5]. The current prominence of Topic 3 supports this shift in research emphasis, indicating that complex robotic interventions are increasingly evaluated through strict methodological frameworks, including RCTs, meta-analysis, extended follow-up and standardized outcome measurement. Importantly, this shift also requires a clearer patient-reported outcome (PRO) framework. Beyond recurrence and perioperative complications, future studies in RHR should assess pain, functional recovery and quality of life by using validated instruments. Generic systems such as PROMIS provide standardized patient-reported assessment across physical, mental and social health domains, whereas hernia-specific tools such as EuraHS-QoL, HerQLes and the Carolinas Comfort Scale provide disease-specific evaluation of abdominal wall function, pain and mesh-related symptoms [66–69]. This thematic transition marks that this field transit from an isolated technical reports toward comparative studies which integrate clinical endpoints, validated PRO instruments and long-term prognostic assessment.

#### Thematic transition three: the rebalancing of the human-machine interface in robotic surgery

The third transition presents a highly profound thematic inversion. Topic 4 exhibits a decline, whereas Topic 6 rises steadily. This divergence suggests that researchers have moved beyond the early fascination with technical parameters and hardware. With widespread robotic platforms adoption, the equipment itself no longer represents the primary bottleneck. Consequently, research prioritizes have shifted back to human operator. The prominent rise of Topic 6 establishes a clear mandate for surgical education. Unresolved learning curves pose substantial risks to patient safety during the adoption of novel system. Therefore, simulator-based objective assessment, AI-based intraoperative surgical video analysis and standardized dual-console mentoring guidelines such as those introduced by SAGES are now recognized as the safeguards for ensuring safety of robotic surgery [55, 61, 62, 70]. Surgeons should pass high-fidelity simulator assessments at first. Subsequently, they should accumulate extensive experience in routine inguinal hernias with highly forgiving anatomy (Topic 5). They progress to more complex abdominal wall reconstructions only after mastering these foundational procedures (Topic 1).

### Implications

These multidimensional insights offer actionable guidance for stakeholders across the healthcare ecosystem. Crucially, the thematic evolution of RHR aligns with established frameworks of value-based surgical care [71, 72]. To achieve sustainable progress, future efforts should be directed toward three interconnected domains: Education, Economics and Evidence.

Regarding surgical education, the traditional apprenticeship model cannot accommodate the unique cognitive demands of robotic surgery. Residents must adhere to standardized training pathways endorsed by SAGES and the EHS [55, 56, 73]. Training programs should mandate a stepwise authorization approach [74].

In terms of health economics, the rapid growth of this topic (Topic 8) necessitates strict scrutiny from healthcare administrators and policymakers. Institutions must avoid indiscriminate adoption of robotic platforms across all hernia types. For routine inguinal hernias, current evidence does not support routine replacement of laparoscopic repair by robotic platforms [5]. In contrast, expensive robotic resources must be strategically allocated to high-risk cohorts, including patients with morbid obesity, massive defects or parastomal hernias. In these complex scenarios, robotic platforms achieve systemic financial balance by reducing severe complications and hospital stays [44, 75, 76].

Finally, regarding clinical evidence, the field increasingly favors comparative studies with stronger methodology over isolated retrospective feasibility reports [66, 77]. Future research should prioritize multicenter randomized designs, pragmatic comparative effectiveness studies and standardized outcome reporting. To establish methodologically sound comparisons, these efforts must be anchored against the historical benchmarks of open and laparoscopic repairs, where recurrence and chronic pain often continue to emerge several years postoperatively [78, 79]. Therefore, it is suggested that researchers conduct studies with 5 to 10 years follow-up periods. This extended duration is essential to confirm the advantages of r-eTEP and rTAR regarding long-term recurrence and chronic pain [80]. Furthermore, exploring artificial intelligence for surgical risk prediction and augmented reality navigation are likely to become important directions for future investigation [81–83].

### Limitations

Although this study utilized advanced machine learning and multidimensional network mapping, inherent bibliometric limitations remain. Firstly, the search was restricted to two databases (WoSCC and Scopus) and only English articles and reviews were included. This linguistic bias may underrepresent regional technological innovations. For instance, pioneering studies evaluating domestic Chinese robotic platforms (KangDuo, MicroPort) or non-English European reports are likely overlooked.

Secondly, topic models derived from the existing literature may underrepresent the most recent and rapidly evolving technological frontiers. In the present study, the emerging SP system is one such example. SP introduces a different access paradigm, distinct training requirements and potentially different economic implications, yet SP-specific hernia literature remains only sparsely represented in our current corpus. Therefore, the present bibliometric framework mainly reflects the historical trajectory of multi-port robotic platforms. Future studies should incorporate single-port evidence to better characterize the evolving anatomical, educational and economic landscape of RHR.

Thirdly, although the LDA model demonstrates robust dimensionality reduction for massive corpora, topic modeling still involves subjective interpretation. The assignment of thematic labels was based on manual review of representative words, which may allow different researchers to group or name topics differently. This limitation may be particularly relevant in RHR, where terms related to surgical technique, risk and outcomes often overlap within the same article.

Fourthly, we were unable to perform a strict bibliometric stratification by hernia type. Although several LDA topics were strongly associated with specific clinical areas, including inguinal hernia repair, ventral or incisional hernia reconstruction and hiatal hernia management, the bibliographic metadata did not provide consistently structured information on hernia subtype. Many studies also included mixed hernia populations or discussed multiple procedures simultaneously. Therefore, our topic-based interpretation should not be regarded as a formal subtype-specific comparison across hernia categories.

Finally, the PCA-based HJ-Biplot should be interpreted with caution. The first two principal components explained 33.14% of the total variance, so the two-dimensional plot cannot fully represent the document-topic matrix. As shown in Supplementary Table 5, higher-order components also explained substantial proportions of variance, indicating that topic variation was distributed across multiple dimensions. Therefore, the four thematic clusters visualized in the biplot should be regarded as conceptual groupings rather than definitive statistical partitions. Overlap between these domains is expected, as many studies in RHR simultaneously involve surgical technique, outcomes, training and resource use.

## Conclusion

This multidimensional bibliometric study reveals a significant thematic transition in RHR. By synthesizing macroscopic descriptive data with advanced machine learning algorithms, our results expose that the research focus has gradually shifted away from early hardware feasibility. Publications focusing on equipment iteration have experienced an observable decline. Instead, rigorous scrutiny under the principles of value-based healthcare currently characterizes the field.

Currently, the academic frontier is governed by a dual mandate. Researchers must simultaneously generate high-level evidence and justify health economics. On the clinical front, technical feasibility alone no longer validates advanced reconstructive techniques (r-eTEP, rTAR). These procedures now require strict assessment through RCTs and long-term follow-up. On the parallel economic front, deploying robotic platforms in high-risk cohorts has promoted a critical demand for precise cost-benefit analysis.

Finally, our panoramic mapping synthesizes the core domains supporting clinical decision-making. At present, the interconnected domains of Evidence, Education and Economics emerge as a foundational framework of this field. Rather than an isolated phenomenon, this thematic convergence aligns with established frameworks of value-based surgical care. Continuous integration of these three domains will be essential to ensure the sustainable advancement of RHR.

## Supplementary Information

Below is the link to the electronic supplementary material.

Supplementary Material 1

Supplementary Material 2

## Data Availability

The datasets generated and analyzed during the current study are derived from publicly available resources. The original literature data were collected from the Web of Science Core Collection (WoSCC) and Scopus databases. The processed data supporting the conclusions of this article are available from the corresponding author upon reasonable request.
