# Supplementary material for: Mapping the scientific landscape of robotic hernia repair: a bibliometric and topic modeling analysis of thematic transitions
Source: J Robot Surg. 2026 Jun 19;20(1):619. doi: 10.1007/s11701-026-03534-y (PMC13282322; doi:10.1007/s11701-026-03534-y)
Supplement: Supplementary file 1 — Supplementary Material 1 [file 11701_2026_3534_MOESM1_ESM.docx]

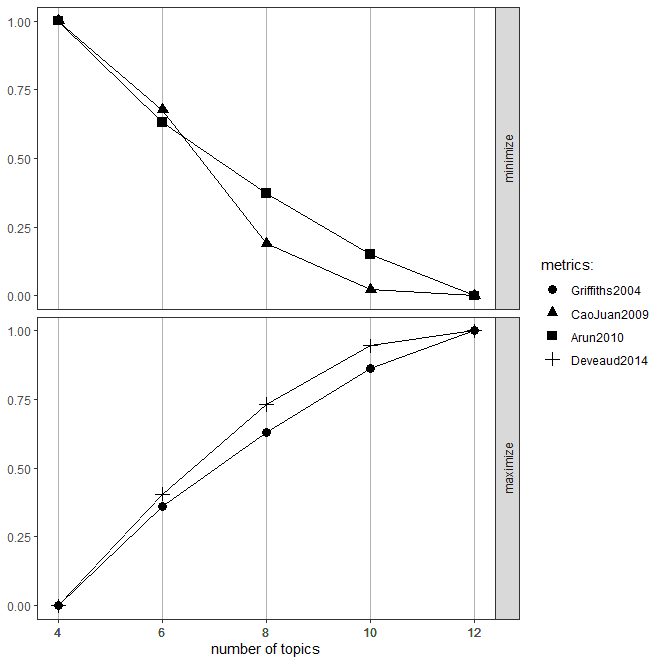


**Supplementary Figure 1. Quantitative diagnostic metrics for LDA topic-number selection**


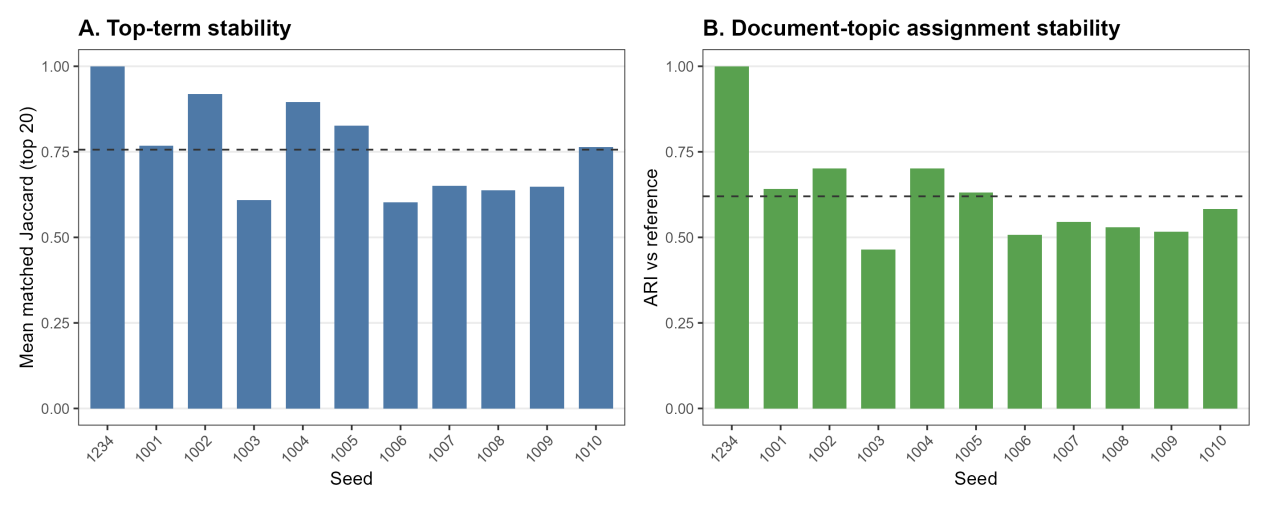


**Supplementary Fig. 2. Random-seed stability of the final k = 8 LDA model. A** Matched top-20 term Jaccard similarity relative to the reference seed. **B** Adjusted Rand index for document-level primary-topic assignment relative to the reference seed. Dashed lines indicate mean values across repeated runs.


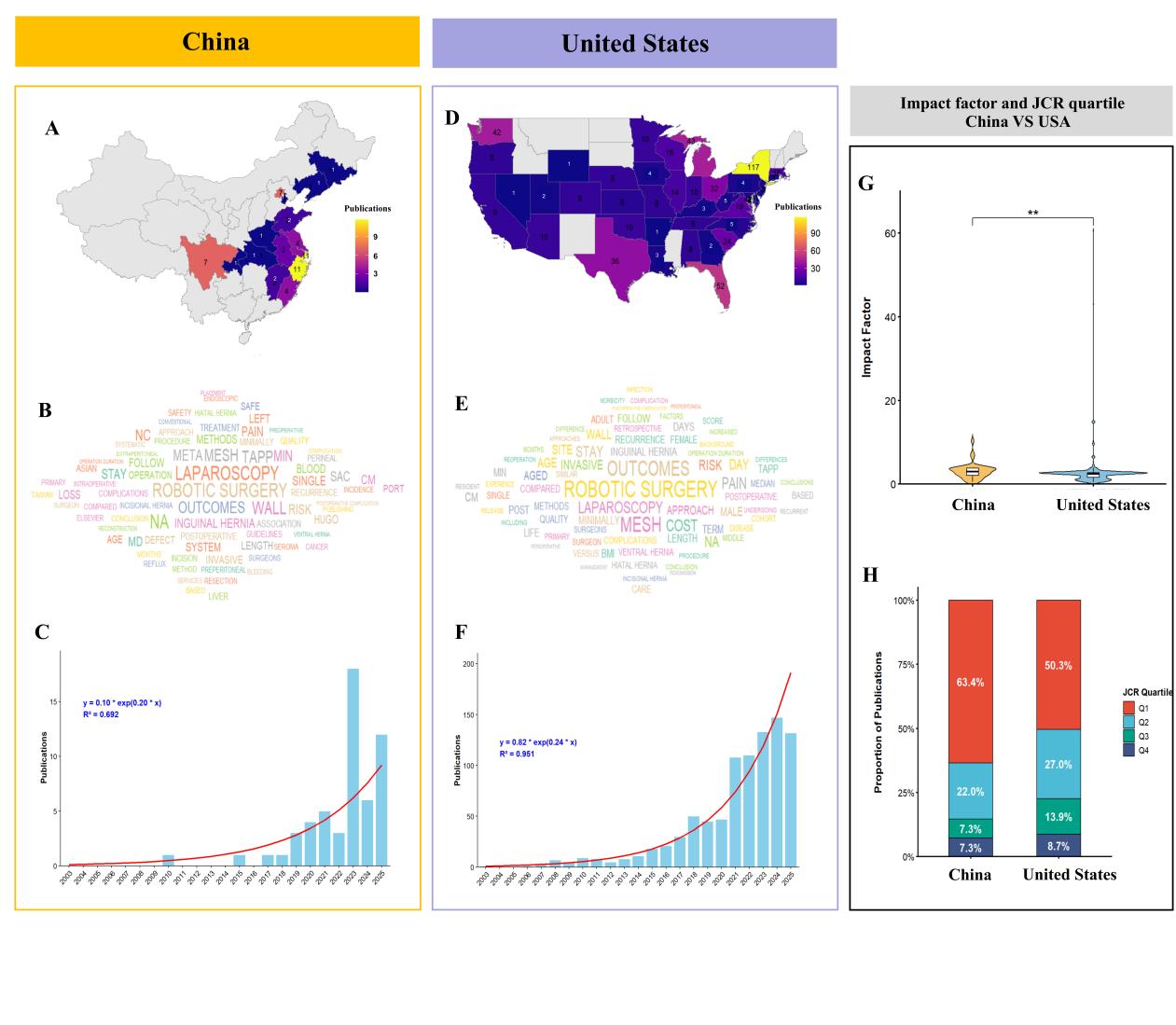


**Supplementary Figure 3. The differences between China and the United States in RHR. A D** The number of publications in different regions of China and the United States. **B** **E** Keyword cloud maps of China and the United States. **C F** The annual publication growth curve of China and the United States. **G H** Comparison of 5-year IF and proportion of JCR quartile between the literature of Chinese and the United States.

**Comparison between China and United States**

To further investigate the development between China and United States, we conducted a comparative analysis. Analysis of publication trajectories reveals a significant divergence (Supplementary Fig. 3). The United States demonstrates a robust exponential growth pattern (y = 0.82×e0.24x, R2=0.951) with a substantial total volume. In contrast, Chinese research output exhibits a slower growth rate (y = 0.10×e0.20x, R2=0.692) and a lower overall baseline. Sub-national geographic mapping highlights distinct spatial distributions within these countries. Research in the United States is widely dispersed across multiple states, with major contributions from regions such as California, Texas, and New York. Conversely, Chinese research is highly centralized in economically developed coastal provinces, with minimal academic output from inland areas.

Furthermore, an assessment of publication quality reveals an interesting statistical paradox. Although the United States produces literature with significantly higher peak impact factors (Supplementary Fig. 3G), Chinese research maintains a more concentrated proportion of top quartile publications. Specifically, 63.4% of Chinese papers are published in JCR Q1 journals, compared to 50.3% for the United States. Finally, keyword co-occurrence analysis reveals a thematic convergence between the two nations. Both the United States and Chinese research paradigms are dominated by identical core terminologies, such as "robotic surgery," "laparoscopy," and "outcomes." While these foundational objectives remain parallel, subtle regional nuances exist within secondary keywords. Specifically, United States literature leans toward economic evaluations including "cost", whereas Chinese research emphasizes specific procedural execution such as "TAPP". Nevertheless, the clinical paradigm across both nations remains tightly aligned.
